# Supplementary material for: The Construction and Exploration of a Comprehensive MicroRNA Centered Regulatory Network in Foxtail Millet (Setaria italica L.)
Source: Front Plant Sci. 2022 May 6;13:848474. doi: 10.3389/fpls.2022.848474 (PMC9121102; doi:10.3389/fpls.2022.848474)
Supplement: Supplementary file 6 [file Data_Sheet_6.zip › Sit-miR398b_Seita.6G251600.1_569_TPlot.pdf]

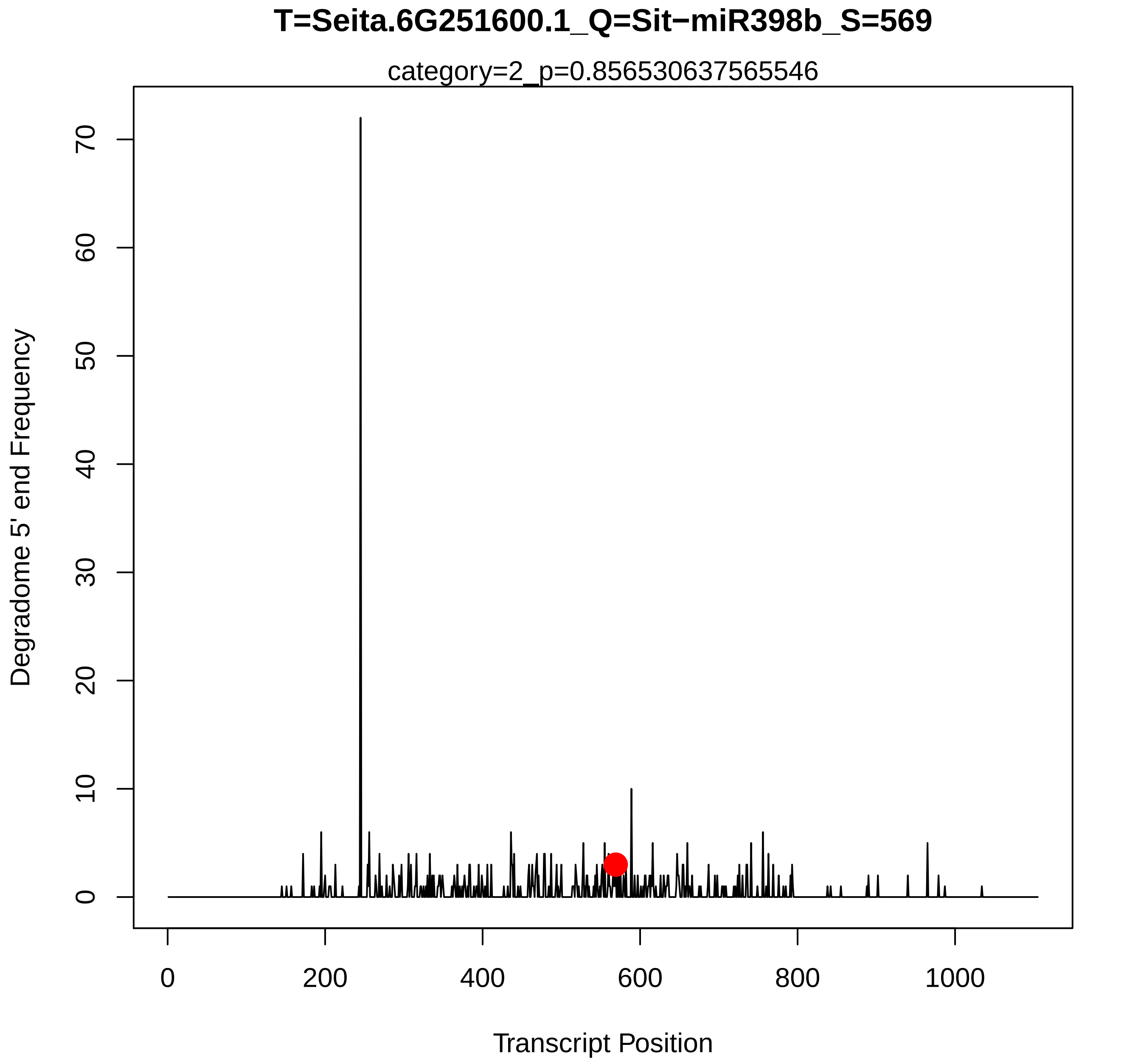

T=Seita.6G251600.1_Q=Sit−miR398b_S=569
category=2_p=0.856530637565546
Degradome 5' end Frequency
0
10
20
30
40
50
60
70
0
200
400
600
800
1000
Transcript Position
